# Domain shuffling of cyclodextrin glucanotransferases for tailored product specificity and thermal stability

**DOI:** 10.1002/2211-5463.12588

**Published:** 2019-01-16

**Authors:** Christian Sonnendecker, Wolfgang Zimmermann

**Affiliations:** ^1^ Department of Microbiology and Bioprocess Technology Institute of Biochemistry Leipzig University Germany

**Keywords:** cyclodextrin glucanotransferase, domain shuffling, large‐ring cyclodextrin, mutagenesis, product specificity, thermostability

## Abstract

Cyclodextrin glucanotransferases (CGTases) convert α‐1,4‐glucans to cyclic oligosaccharides (cyclodextrins, CD), which have found applications in the food and the pharmaceutical industries. In this study, we used two CGTases with different cyclization activities, product specificities, and pH and temperature optima to construct chimeric variants for the synthesis of large‐ring CD. We used (a) a synthetic thermostable CGTase mainly forming α‐ and β‐CD (CD6 and CD7) derived from *Geobacillus stearothermophilus* ET1/NO2 (GeoT), and (b) a CGTase with lower cyclization activity from the alkaliphilic *Bacillus* sp. G825‐6, which mainly synthesizes γ‐CD (CD8). The A1, B, A2, and CDE domains of the G825‐6 CGTase were replaced with corresponding GeoT CGTase domains by utilizing a megaprimer cloning approach. A comparison of the optimum temperature and pH, thermal stability, and CD products synthesized by the variants revealed that the B domain had a major impact on the cyclization activity, thermal stability, and product specificity of the constructed chimera. Complete suppression of the synthesis of CD6 was observed with the variants GeoT‐A1/B and GeoT‐A1/A2/CDE. The variant GeoT‐A1/A2/CDE showed the desired enzyme properties for large‐ring CD synthesis. Its melting temperature was 9 °C higher compared to the G825‐6 CGTase and it synthesized up to 3.3 g·L^−1^ CD9 to CD12, corresponding to a 1.8‐ and 2.3‐fold increase compared to GeoT and G825‐6 CGTase, respectively. In conclusion, GeoT‐A1/A2/CDE may be a candidate for the further development of CGTases specifically forming larger CD.

**Abbreviations**
CD: cyclodextrins
CGTase: cyclodextrin glucanotransferase
HPAEC‐PAD: high‐pressure anion exchange chromatography with pulsed amperometric detection
nanoDSF: nano differential scanning fluorimetry

Cyclodextrin glucanotransferases (CGTases) convert α‐1,4‐glucans to cyclic oligosaccharides (cyclodextrins, CD) 1, 2. CD can form reversible complexes with guest molecules and have found applications mainly in the food and the pharmaceutical industries 3. With starch as substrate, CGTases produce a mixture of CD of various ring sizes mostly composed of 6, 7, or 8 glucose units (CD6, CD7, CD8). The preferential CD product formed is dependent on the type and origin of the CGTase 4. Only few CGTases synthesize CD8 as the main CD product and with lower turnover rates compared to CGTases predominantely forming CD6 and CD7. CD8 displays interesting properties for potential applications such as a larger cavity size compared to CD6 and CD7, higher water solubility, and bioavailability 5. CD9 to CD12 are formed as side products in small amounts during the initial cyclization reaction, followed by a subsequent conversion to smaller ring sizes 6. Their complex‐forming abilities have not been studied in any detail due to their low production yields and tedious downstream processing required for their isolation 7, 8, 9, 10, 11. CGTases are composed of five domains, A, B, C, D, and E, where domain A forms a (β/α)_8_ barrel with a prominent and extensive loop, the B domain, between the β sheet 3 and helix 3 of the A domain to separate this domain into A1/B/A2 12. The A/B or A1/B/A2 structure is conserved in the α‐amylase family and represents the main substrate binding site and catalytic center of the enzyme 13. The domains C and E contain further glucan binding sites and probably guide the glucan substrate chain toward the active site, whereas domain D has been suggested to participate in the positioning of the domain E 14, 15.

Three conserved carboxylates located in the A2 domain catalyze the conversion of starch to CD by an α‐retaining double displacement mechanism in which a glucan is covalently bound and subsequently cleaved. The intermediate is then transferred to the C4‐OH group of its reducing end to form a CD in an intramolecular transglycosylation reaction 16. The cleavage of the glucan chains occurs between the glucan binding subsites −1 and +1. Glucose residues toward the reducing end (acceptor site) are numbered positively and residues toward the non‐reducing end (donor site) negatively 17. Besides the cyclization, three further intermolecular transfer reactions are also catalyzed by CGTases. The coupling reaction is the reversal of the cyclization reaction, where a CD is cleaved and transferred to a linear glucan acceptor. The disproportionation reaction describes the transfer of a linear glucan fragment to another linear glucan. In a hydrolysis reaction, water can also act as an acceptor for the glucan intermediate 18.

With the aim to increase the yield of larger CD and the thermostability of the G825 CGTase, a PCR‐based domain shuffling between a CGTase derived from the mainly CD8‐forming alkaliphilic *Bacillus* sp. G825‐6 (G825 CGTase) and a thermostable CGTase from *Geobacillus* *stearothermophilus* 19, 20 was performed. Four segments encoding for the A1, B, A2, and CDE of the CGTases were shuffled, and chimeric variants were characterized with regard to their CD product spectrum, thermal stability, and temperature and pH optima.

## Materials and methods

### Construction of chimeric CGTase expression vectors

**Figure 1:**
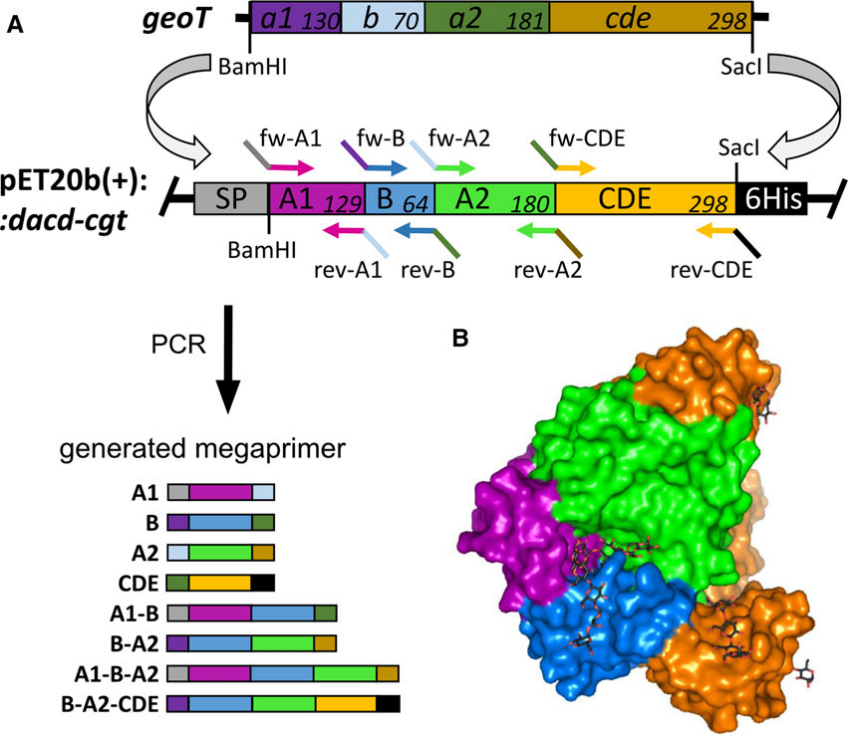
Construction of chimeric vectors. The color code corresponds to the protein domains of the respective CGTases. (A) The synthetic *geoT* gene was cloned into the pET20b(+)::*dacD* vector. Domain lengths on amino acid level for the *geoT* and cgt gene and primer binding sites are shown. Megaprimers were generated by PCR. (B) Surface model of the G825‐6 CGTase showing the position of the domains.

A previously constructed CGTase expression vector pET20b(+)::*dacD‐cgt* encoding for the 671 amino acids of the mature G825 CGTase (Fig. S1) and the pelB sequence substituted with dacD was used for the expression 21. A synthetic DNA fragment (*geoT*) (Fig. S2) encoding for the 679 amino acid of the mature CGTase derived from the *G. stearothermophilus* ET1 CGTase 20 with substitutions S33T, E64D, N180S, K204R, L214I, V241I, F266Y, S400A, A457G, A471S, N609D, L617M, and I654T was cloned into the expression vector pET20b(+)::*dacD‐geoT* between BamHI and SacI restriction sites, as illustrated in Fig. 1 21, 22. A domain rearrangement was achieved by substituting the DNA regions encoding for the GeoT domains A1, B, A2, and CDE with the corresponding G825‐6 fragments. Therefore, a set of eight primer pairs were used to generate eight megaprimers which encode for the G825‐6 CGTase domains A1, B, A2, and CDE (domains C, D, and E were considered as one structural unit), as well as flanking domain megaprimers A1‐B, B‐A2, A2‐CDE, A1‐B‐A2, B‐A2‐CDE (Fig. 1). Primers used for the construction of chimeric variants by restriction‐free cloning are shown in Fig. S3 23. The megaprimer PCR products were purified by agarose gel electrophoresis and used as megaprimers for a second PCR with pET20b(+)::*dacD‐geoT* or other chimeric vectors as template to construct the variants. *Escherichia coli* XL10Gold cells were transformed with the DpnI‐digested PCR products. The constructed plasmids were then isolated and sequenced prior to their introduction into *E. coli* BL21 (DE3). To designate the chimeric vectors and proteins, the term GeoT plus the substituted domain from the G825‐6 CGTase was used.

### Enzyme production, purification, and characterization

*Escherichia coli* BL21 (DE3) with recombinant expression vectors encoding for the G825‐6 CGTase and GeoT CGTase, as well as 14 chimeric variants, were expressed and extracellular fractions were purified and analyzed as described previously 21.

### Colorimetric assay for the formation of CD7

To determine pH and temperature optima of the CGTases, a phenolphthalein assay for the selective detection of CD7 was used 24 with the following modifications: 100 μL of a 20 g·L^−1^ soluble starch solution in CGTase buffer was incubated with the CGTase for 10 min followed by a 10‐min heat inactivation at 95 °C. After cooling to room temperature, 600 μL phenolphthalein reagent was added. After mixing, 175 μL was transferred to a 96‐well microtiter plate for spectroscopic analysis at 553 nm with a 96‐well microplate spectrophotometer (PowerWave XS, Bio Tek, Winooski, VT, USA). A calibration curve was constructed in the range between 0.1 and 1 g·L^−1^ CD7. An appropriate amount (0.04–0.6 μg) of CGTase was added to synthesize between 0.6 and 0.8 mg CD7 within the reaction time of 10 min at optimum reaction conditions. For the determination of the temperature optimum of the CGTases, 100 μL starch substrate was incubated with the enzymes at 40 °C, 50 °C, 60 °C, 70 °C, 80 °C, 90 °C, and 100 °C. The pH optimum was determined by performing the synthesis reactions in 25 mm buffer (acetic acid buffer pH 4–5, maleate buffer pH 6, phosphate buffer pH 7, Tris/HCl buffer pH 8–9, and glycine‐NaOH buffer pH 10–11) containing 20 g·L^−1^ starch, 10 mm KCl, and 5 mm MgCL_2_.

### Determination of the melting temperature of the CGTases

Melting temperatures (*T*_m_) were determined by nano differential scanning fluorimetry (nanoDSF) (Prometheus NT.48; Nanotemper Technologies, Munich, Germany) based on a tryptophane fluorescence ratio 350/330 nm. The protein denaturation curves were determined in a range between 20 °C and 95 °C with a slope of 1 °C·min^−1^. Melting temperatures (*T*_m_) were calculated as the inflection point of the denaturation curve by first derivate analysis.

### CD synthesis and analysis

For determination of the CD produced by the CGTases, 20 g·L^−1^ soluble starch in CGTase buffer (25 mm Tris/HCl, 25 mm maleate, 10 mm KCl, 10 mm MgCL_2_, pH 6 for GeoT, pH 7 for chimeric variants, and pH 8.5 for the G825‐6 CGTase) was boiled and cooled prior to the addition of 0.2 μg purified CGTase. Of the GeoT CGTase, 0.05 μg was added, and of the GeoT‐B/CDE CGTase, 1 μg was added. Synthesis reactions were performed in 1 mL total volume at 50 °C (GeoT at 60 °C), and 100 μL of aliquots was removed after 1, 4, 8, 16, and 24 h (with GeoT also after 48 and 72 h) and added to 100 μL 0.2 m acetic acid buffer, pH 4.5, followed by heat inactivation and glucoamylase treatment as previously described 25. Samples were diluted and analyzed by high‐pressure anion exchange chromatography with pulsed amperometric detection (HPAEC‐PAD) as described previously 25. Elution was performed at −10 to 2 min with 8 mm NaNO_3_, followed by linear gradients to 14, 22, 36, 80, 200, 100, and 8 mm NaNO_3_ at 10, 35, 45, 55 57, 58, and 62 min after injection. For the determination of Michaelis–Menten kinetics, 0.2 μg GeoT CGTase in 1 mL volume was incubated at 60 °C for 30 min with 1, 2, 4, 6, 8, 10, 15, and 20 g·L^−1^ soluble starch in CGTase buffer, pH 6. For the G825‐6 CGTase, a reaction temperature of 50 °C and a reaction time of 60 min at pH 8.5 were used 26. Samples were analyzed by HPAEC‐PAD accordingly. Calibration curves with authentic standards were constructed for the determination of CD concentrations.

### Protein alignments and modeling

Pairwise and multiple protein alignments were performed with the EMBOSS‐NEEDLE and MUSCLE algorithm from the EMBL‐EBI web service 27. Mature protein sequences for the G825‐6 CGTase (GenBank: BAE87038), *Bacillus circulans* CGTase (PDB: 1EO5), and *G. stearothermophilus* CGTase NO2 (UniProtKB: P31797) and ET1 (GenBank: AAD00555) were used. Models were generated with the SWISS‐MODEL web server 28. The PDB structure 1CYG of the CGTase from *G. stearothermophilus* isolate NO2 19 was used as a template to generate the GeoT model structure. The PDB structure 4JCM from the *Bacillus clarkii* CGTase was used as a template for the G825‐6 CGTase model. The pymol software (Molecular Graphics System, New York, NY, USA; Version 0.99 Schrödinger, LLC) was used for the visualization of the CGTase models with superimposed substrate with PDB 1CXK 29.

## Results

### Construction and expression of the chimeric CGTase variants

**Figure 2:**
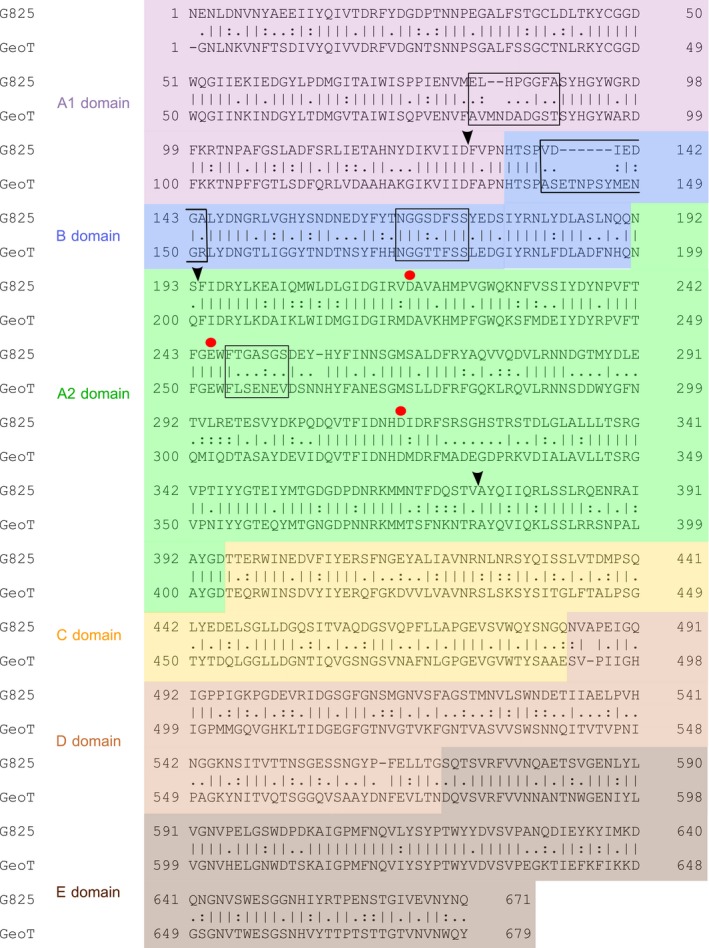
Pairwise sequence alignment of the mature G825‐6 CGTase and the GeoT CGTase. Red circles mark the catalytic triad. An arrow indicates the division into segments used in this study. Boxed sequences correspond to regions with a greater variation in the backbone (Fig. 7). Other symbols: (I) matches, (‐) gaps, (.) mismatches scoring 0.5 or less in the Gonnet PAM 250 matrix 27, (:) mismatches scoring more than 0.5 in the Gonnet PAM 250 matrix. The alignment was created using the EMBOSS‐NEEDLE algorithm from the EMBL‐EBI web service 27.

Protein sequence alignments were generated to calculate the sequence identity between the mature CGTase sequences from G825‐6, GeoT, *G. stearothermophilus* ET1 and NO2, and the synthetic GeoT (Table 1). A sequence alignment of the G825 and GeoT sequences is shown in Fig. 2. The GeoT sequence was derived from the ET1 CGTase. By sequence comparison of the CGTase protein sequences from *G. stearothermophilus* ET1, NO2, and G825‐6, the sequence identity between the GeoT and G825‐6 CGTases was increased from 58.7% to 60.2% by inserting 13 amino acid substitutions. Therefore, resides of the ET1 sequence which showed mismatches with the NO2 sequence were exchanged where the NO2 and G825‐6 sequences showed a match at corresponding positions. Eight megaprimers that encode for single or flanking G825‐6 CGTase domains (Fig. 1A) were synthesized in a first PCR and used in a second PCR for their introduction into the *geoT* expression vector. To construct chimeric vectors with two domains not flanking each other, a first generation of *geoT* chimeric vectors was necessary due to the recognition sites of the primers. To create the variant GeoT‐A1/CDE, the chimeric *geoT*‐A1 vector was used as a template in the second PCR with a CDE megaprimer encoding for the G825‐6 CGTase CDE domains. Chimeric vectors were sequenced, and respective proteins were recombinantly expressed, purified, characterized, and compared to their template CGTases. A surface model of the G825‐6 CGTase domains is shown in Fig. 1B. The five variants GeoT‐A2, A1/A2, B/A2, A2/CDE, and B/A2/CDE showed no enzyme activity after expression.

**Table 1:** DNA sequence comparison of the CGTases. (A) The percent sequence identity between the G825‐6 CGTase, the synthetic GeoT CGTase, and *Geobacillus stearothermophilus* CGTase derived from strains NO2 and ET1 is shown. (B) Percent sequence identity and number of gaps between the GeoT CGTase and the corresponding G825‐6 CGTase domain fragments used in this study

A)					(B)		
CGTase sequence identity (%)					Domain	Identity (%)	Gaps
G825‐6	100	58.74	60.24	58.74	A1	64.1	2
NO2	58.74	100	90.57	88.66	B	57.1	6
GeoT	60.24	90.57	100	98.09	A2	60.8	1
ET1	58.74	88.66	98.09	100	CDE	56.6	3

### Temperature and pH optimum of the CGTase variants

**Figure 3:**
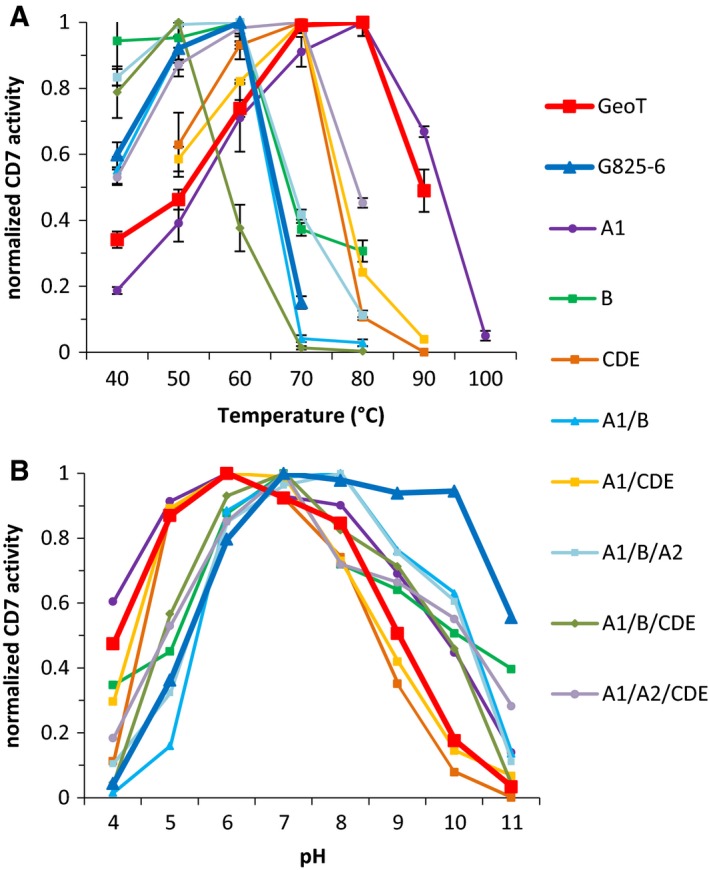
Optimum temperature (A) and pH (B) of the G825‐6 CGTase, the GeoT CGTase, and the chimeric variants for the synthesis of CD7. The data are normalized to the maximum activity of each enzyme. Mean values (*N* = 3) are shown; SD is indicated in (A).

The optimum reaction temperature of the CGTases for the synthesis of CD7 was determined in a range between 40 °C and 90 °C (Fig. 3A). The GeoT CGTase showed a temperature optimum between 70 °C and 80 °C and the G825‐6 CGTase between 50 °C and 60 °C. High‐temperature optima were observed for the chimeric variants GeoT‐A1 (80 °C), CDE and A1/CDE (70 °C), and A1/A2/CDE (60–70 °C), respectively. Lower temperature optima were observed for GeoT‐A1/B (60 °C), A1/B/A2 and B with 50–60 °C, and A1/B/CDE 50 °C. The GeoT CGTase showed a pH optimum between pH 5 and 7, and the G825‐6 CGTase was most active between pH 8 and 10 (Fig. 3B). The GeoT‐A1 and CDE variants showed a pH optimum between pH 5 and 7, similar to the GeoT CGTase, whereas the other chimeric variants were most active between pH 6 and 8.

### Thermal stability of the GeoT and G825‐6 CGTases and the chimeric variants

**Figure 4:**
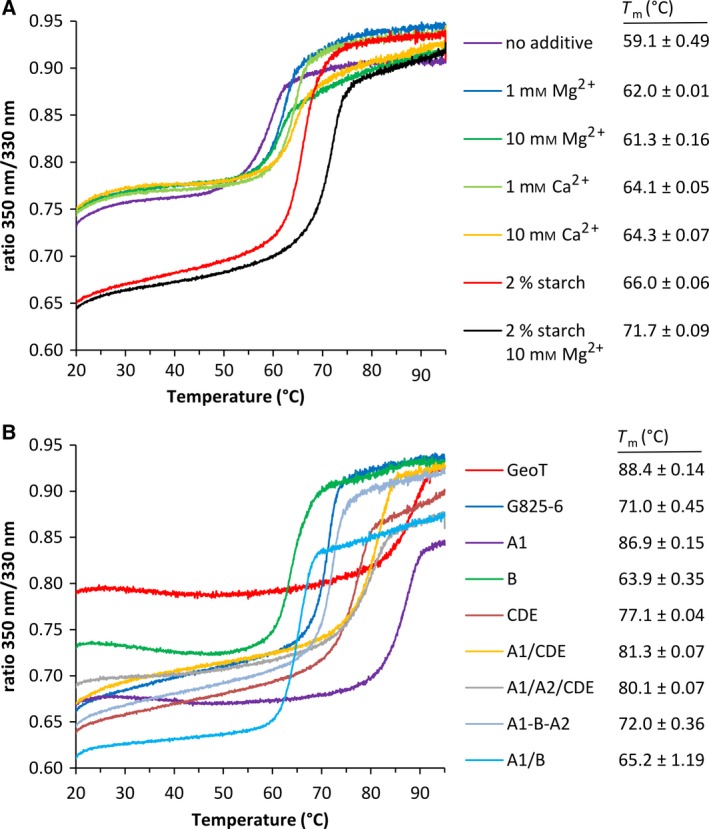
Determination of CGTase thermal stability. (A) Thermal stability of the G825‐6 CGTase in the presence of additives. (B) Analysis of the thermal stability of the G825‐6 and the GeoT CGTases and the chimeric variants by nanoDSF. Mean values (*N* = 3) were used to construct the curves; mean values (*N* = 3 ± SD) of the *T*_m_ are also shown.

The effect of additives on the melting temperature (*T*_m_) of the G825‐6 CGTase was determined to evaluate their influence on the stability of the CGTases (Fig. 4A). The *T*_m_ increased from 59 °C to 62 °C in the presence of 1–10 mm MgCl_2_ and by 5 °C in the presence of 1–10 mm CaCl_2_. The addition of starch increased the *T*_m_ to 66 °C and further addition of 10 mm MgCl_2_ resulted in a *T*_m_ of 71.7 °C. The *T*_m_ of all the CGTase variants was then determined in the presence of 20 g·L^−1^ starch and 10 mm MgCl_2_ (Fig. 4B). The parental GeoT and the GeoT‐A1 chimera showed the highest *T*_m_ with 88 °C and 87 °C, respectively. The variants A1/CDE and A1/A2/CDE displayed a *T*_m_ of 81 °C and 80 °C, respectively, while the CDE chimera showed an intermediate *T*_m_ of 77 °C. The chimeras including the B domain of the G825‐6 CGTase showed lower *T*_m_ values. The variants A1/B/A2, A1/B, and variant B displayed a *T*_m_ of 72 °C, 65 °C, and 64 °C, respectively.

### Yield and size spectra of CD synthesized by the GeoT and G825‐6 CGTases, and the chimeric variants

**Figure 5:**
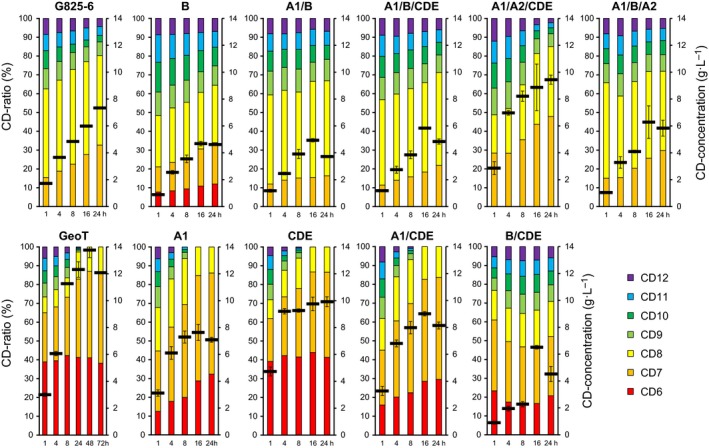
CD synthesized by the G825‐6 CGTase, the GeoT CGTase, and the chimeric variants. The ratios of the different CD in percent and the total amounts of CD8 to CD12 in g·L^−1^ synthesized during a reaction time of 1–24 h at 50 °C (black bars, *N* = 3 ± SD) determined by HPLC are shown. 0.2 μg purified CGTase was added per reaction, except for GeoT (50 ng) and GeoT‐B/CDE (1 μg).

The GeoT CGTase showed higher turnover numbers for the cyclization reaction to synthesize CD6 to CD12 compared to the G825‐6 CGTase (Table 2). For a comparison of the ratios of the CD synthesized by the two CGTases and the variants, we selected reaction time, reaction temperature, and protein concentrations where all proteins were active and could be compared. The GeoT CGTase synthesized CD6 and CD7 as the main products, while only small amounts of CD8 and larger CD were produced. CD9 and the larger CD were subsequently degraded during longer reaction times (Fig. 5). The G825‐6 CGTase synthesized primarily CD8 as well as some CD7 and larger CD. However, both enzymes showed similar maximum yields of 0.6 g·L^−1^ CD9 and 0.4 g·L^−1^ CD12, respectively.

**Table 2:** Michaelis–Menten kinetics of the cyclization reaction of the GeoT and the G825‐6 CGTase. *K*_m_ and *k*_cat_ values for the synthesis of CD6 to CD12. Data were analyzed by non‐linear regression. *N* = 3 ± standard error

GeoT	*K*_m_ (g·L^−1^)	*k*_cat_ (s^−1^)	G825‐6^a^	*K*_m_ (g·L^−1^)	*k*_cat_ (s^−1^)
CD6	3.3 ± 0.48	188.9 ± 9.41	CD6	–	–
CD7	4.8 ± 0.78	127.9 ± 7.43	CD7	4.1 ± 0.66	21.9 ± 1.27
CD8	4.8 ± 0.73	39.0 ± 2.12	CD8	5.1 ± 0.84	65.0 ± 4.23
CD9	6.5 ± 0.85	28.7 ± 1.53	CD9	4.0 ± 0.75	11.3 ± 0.75
CD10	8.0 ± 1.58	26.4 ± 2.31	CD10	3.6 ± 0.67	7.9 ± 0.50
CD11	8.3 ± 0.98	22.7 ± 1.21	CD11	4.3 ± 0.80	7.8 ± 0.53
CD12	8.6 ± 1.79	19.0 ± 1.81	CD12	4.6 ± 0.83	7.1 ± 0.48

a Data from Ref. 26.

The CD size spectrum synthesized by the GeoT CGTase was not influenced by the replacement of the CDE fragment with the corresponding G825‐6 CGTase sequence. However, substitutions of A1 and A1/CDE doubled the proportion of CD8 synthesized after 1 h of reaction, while less CD6 was formed (Fig. 5). These variants showed high initial activity; however, less total CD amount was formed in a later stage of the reaction. The variants GeoT‐B and GeoT‐B/CDE showed a CD size spectrum similar to G825‐6; however, they still also synthesized CD6 and their overall cyclization activity was reduced. The variants GeoT‐A1/B and GeoT‐A1/B/CDE were also similar to the G825‐6 CGTase, except that a lesser total amount of CD was produced and the proportion of CD7 was lower. The variant A1/A2/CDE synthesized twice as much CD after a reaction time of 1 h compared to the G825‐6 CGTase. After a reaction time of 24 h, 47% of the soluble starch substrate was converted to CD7 to CD12. A maximum yield of CD7 (4.5 g·L^−1^) and CD8 (3.9 g·L^−1^) was obtained after 24 h. The maximum yields of CD9 (1 g·L^−1^), CD10 (0.9 g·L^−1^), CD11 (0.8 g·L^−1^), and CD12 (0.7 g·L^−1^) were obtained after 4 h of reaction. All of the chimeric CGTases produced lower total amounts of CD compared to the GeoT CGTase.

## Discussion

To engineer a thermostable CGTase synthesizing CD8 and larger CD in high yields, we combined the CD8 product specificity of the G825‐6 CGTase with the high cyclization activity and thermostability of the GeoT CGTase. The larger CD have been previously available only in very limited amounts and could find novel applications in supramolecular chemistry 4. Thermostability is a further desired property of a CGTase since a higher reaction temperature reduces the viscosity of the starch substrate and can result in higher yields of CD.

**Figure 6:**
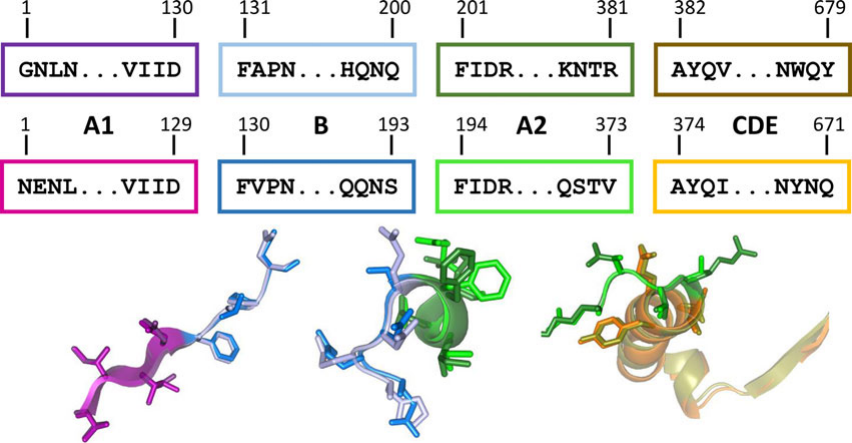
Intersections used for the domain shuffling are shown with four flanking amino acid residues and the corresponding numbering for the mature CGTases for domains A1, B, A2, and CDE as well as structure models of the intersection regions.

**Figure 7:**
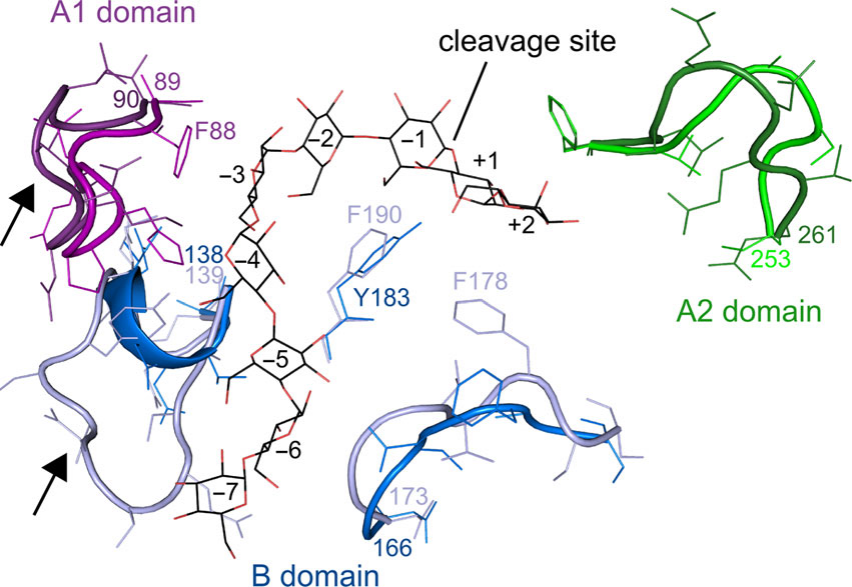
Model of the main substrate binding sites of the CGTases. Backbone conformations with significant differences between the G825‐6 and the GeoT CGTase are shown as cartoons and side chains as lines. The colors correspond to the domain colors used in Fig. 1. A maltononaose substrate is shown in black lines, and numbers refer to the glucan binding subsites. The substrate winds around a centrally located Y183 (G825‐6) or F190 (GeoT) residue. Arrows indicate the extended loops of the G825‐6 and GeoT CGTases.

The sequence identity between the GeoT and G825‐6 CGTases was adapted to delimit the numbers of positions contributing to the properties of the shuffled CGTases. For the NO2 and ET1 CGTase which share the same properties, the exchange of mismatched residues did not show any effect. To reduce the number of chimeric combinations and to increase the probability of obtaining active chimeras, the CGTase‐specific domains C, D, and E of both structures were treated as one segment 15. The CDE segment also included the C‐terminal helix of the A2 domain to allow the transfer of the complete hinge region and the amino acid residues in its vicinity (Fig. 6). The chimeric variants could be successfully constructed using megaprimers without the need to incorporate restriction sites within the template sequences. In contrast to the parental GeoT CGTase, the substitution of the A1 domain with the G825‐6 CGTase A1 domain resulted in a higher production of CD8 and a lesser formation of CD6 (Fig. 5). The residue T44 from the G825‐6 CGTase A1 domain could be one of the key positions of the A1 domain contributing to the formation of larger CD. Mutagenesis studies have previously indicated a shift from CD7 to CD8 in a similar variant of a CD7‐forming CGTase, presumably due to a space‐gaining effect at the substrate binding subsite −3 30. We further compared the backbone structures of both parental CGTases as well as regions with an altered conformation of their main binding sites (Fig. 7). The loop structure 82–89 of the G825‐6 A1 domain is shorter by two residues compared to the GeoT model structure and contains the residue F88, oriented toward the substrate at the binding subsite −3. This residue is also present in other CD8‐forming CGTases 31 and could be responsible for the observed shift in CD product size specificity. The variant GeoT‐A1 maintained the thermostability of the parental enzyme, indicating that the A1 domain of the G825‐6 CGTase also confers thermostability properties 32.

The strongest effect regarding the temperature optimum, catalytic efficiency, and product specificity of the CGTases was obtained by substituting the B domain. A previous study has reported an increase in the thermostability of a CGTase by replacing the two residues N188D and K192R of the *B. circulans* CGTase within the B domain and associated this domain with the thermal stability of the CGTase 33. Both G825‐6 and GeoT CGTases display the residues D and R at the corresponding positions; however, the thermostability of the chimeric variants was manipulated by substituting the B domain. Therefore, these residues were not responsible for the observed effect in the G825‐6 CGTase.

The loop 139–151 from the GeoT CGTase could form a subsite for the binding of glucose at the position −7. In contrast, the corresponding loop 138–144 in the G825‐6 CGTase is shorter, a typical feature of CD8‐forming CGTases 31. It has been shown that the shorter loop contributed to the CD8‐forming specificity and resulted in a decreased cyclization activity in CD7‐forming CGTases 30, 34. However, the variant GeoT‐B could still synthesize CD6. Apart from that, the GeoT‐B chimeras were similar to the G825‐6 CGTase. The chimera GeoT‐A1/B could not synthesize CD6, which further underscores the importance of the A1 domain for the CD product size specificity. Interestingly, the variant GeoT‐A1/A2/CDE, which corresponded to the G825‐6 CGTase with the B domain from GeoT, showed a higher temperature optimum, a higher CD synthesis activity, and a higher CD7 product share compared to G825‐6. The variant A1/A2/CDE was also not capable of synthesizing CD6. This was apparently due to the presence of the A1 and A2 domains from G825‐6, since only variants with the replacements A1/B and A1/A2 lost the ability to synthesize CD6. A substitution of the CDE segment showed only a small decrease in the total amount of CD synthesized (Fig. 5). The GeoT variants with a substituted A2 domain could not be expressed successfully. The differences in length of both A2 domains were suspected to have resulted in a wrong orientation of the catalytic triad residues. However, inserting N257Ins to adapt the sequence length in the G825‐6 A2 domain of these chimeras did not regain the activity (Fig. 2). A similar effect has been observed previously with the expression of chimeric CGTases, and conformational changes or proteolytic digestion has been suggested as possible explanations 19.

In contrast to previous studies which produced chimeras using template CGTases with higher sequence identity and rather similar properties, we used two template CGTases with significant differences in their pH and temperature optima, cyclization activity, product size spectrum, and sequence length 15, 19, 35, 36. The limitations of site‐specific silent mutations to introduce restriction sites had resulted in a significant loss of activity of CGTase chimeras generated previously 15. In contrast, the PCR‐based approach we employed allowed an efficient sequence‐independent rational shuffling of the desired segments. Earlier studies on CGTase chimeras suggested that the A/B domains were responsible for the CD product size specificity, cyclization activity, and thermal stability 19, 35, 36. Therefore, we decided to shuffle domains A1, B, and A2 separately and thereby divided the N‐terminal part into three segments. We considered the C‐terminal part including the domains C, D, and E as one segment since a correct combination of the domains C, D, and E has been demonstrated to be important for the overall functionality of the CGTase 15. Domain E is supposed to guide the glucan substrate toward the active site of the CGTase, and a wrong positioning of domain E may disturb this process. Furthermore, the segment CDE may help to ensure in particular longer glucan chains to be properly delivered to the active site for processing 18.

A combination of the G825‐6 CGTase domains A1 and B or A1 and A2 was necessary to suppress the synthesis of CD6 by the GeoT CGTase demonstrating that several regions of the G825‐6 CGTase binding site contributed to this feature. The variant GeoT‐A1/A2/CDE could be a candidate for the further development of CGTases specifically forming larger CD. To this end, the B domain of the variant could be gradually adapted to the G825‐6 sequence by a rational design strategy.

## Conflict of interest

The authors declare no conflict of interest.

## Author contributions

CS and WZ conceived and designed the project; CS acquired and analyzed the data; CS and WZ wrote the paper.

## Supporting information


**Fig. S1.** Codon‐optimized sequence encoding for the mature G825‐6 CGTase.
**Fig. S2.** Codon optimized sequence encoding for the mature GeoT CGTase.
**Fig. S3.** Primer sequences selected for domain shuffling. Primer designations refer to the corresponding megaprimer products from the first PCR encoding for G825‐6 CGTase domains. G825‐6 CGTase encoding regions are marked in grey, vector elements of pET20b(+)::dacD are underlined. Unmarked sequences are derived from the geoT fragment and represent target sites for the incorporation of the megaprimer into the vector pET20b(+)::dacD‐geoT in the second PCR.
