# Supplementary material for: Domain shuffling of cyclodextrin glucanotransferases for tailored product specificity and thermal stability
Source: FEBS Open Bio. 2019 Jan 16;9(2):384–95. doi: 10.1002/2211-5463.12588 (PMC6356159; doi:10.1002/2211-5463.12588)
Supplement: Supplementary file 1 — Fig. S1. Codon‐optimized sequence encoding for the mature G825‐6 CGTase. Fig. S2. Codon optimized sequence encoding for the mature GeoT CGTase. Fig. S3. Primer sequences selected for domain shuffling. Primer designations refer to the corresponding megaprimer products from the first PCR encoding for G825‐6 CGTase domains. G825‐6 CGTase encoding regions are marked in grey, vector elements of pET20b(+)::dacD are underlined. Unmarked sequences are derived from the geoT fragment and represent target sites for the incorporation of the megaprimer into the vector pET20b(+)::dacD‐geoT in the second PCR. [file FEB4-9-384-s001.docx]

Supplementary information

Domain shuffling to taylor the product specificity and thermal stability of cyclodextrin glucanotransferases

Christian Sonnendecker^1^, Wolfgang Zimmermann^1*^

1 Dept. of Microbiology and Bioprocess Technology, Institute of Biochemistry, Leipzig University, Johannisallee 23, 04103 Leipzig, Germany

* Corresponding author

E-Mail: wolfgang.zimmermann@uni-leipzig.de

AATGAAAATCTGGATAACGTGAACTATGCGGAAGAAATCATTTATCAGATCGTGACGGATCGCTTTTATGATGGCGATCCGACGAATAATCCGGAAGGCGCGCTGTTTAGCACGGGCTGCCTGGATCTGACGAAATATTGTGGCGGCGATTGGCAGGGCATTATCGAAAAAATCGAAGATGGCTATCTGCCGGATATGGGCATTACGGCGATTTGGATTAGCCCGCCGATTGAAAATGTGATGGAACTGCATCCGGGCGGTTTTGCGAGCTATCATGGCTATTGGGGCCGTGATTTTAAACGCACGAATCCGGCGTTTGGCAGCCTGGCCGATTTTAGCCGCCTGATTGAAACGGCGCATAACTATGACATCAAAGTGATCATCGATTTTGTGCCGAATCATACGAGCCCGGTGGATATTGAAGATGGCGCGCTGTATGACAATGGCCGCCTGGTGGGCCATTATAGCAACGACAACGAAGATTATTTTTATACGAACGGCGGCAGCGATTTTAGCAGCTATGAAGATAGCATCTATCGCAATCTGTATGATCTGGCCAGCCTGAATCAGCAGAATAGCTTTATCGATCGCTATCTGAAGGAAGCGATTCAGATGTGGCTGGATCTGGGCATTGATGGCATTCGCGTGGATGCGGTGGCGCACATGCCGGTGGGCTGGCAGAAAAATTTTGTGAGCAGCATCTATGATTATAATCCGGTGTTTACGTTTGGCGAATGGTTTACGGGCGCGAGCGGCTCTGATGAATACCATTATTTTATCAACAATAGCGGCATGAGCGCGCTGGATTTTCGCTATGCGCAGGTGGTGCAGGATGTGCTGCGCAATAATGATGGCACGATGTATGACCTGGAAACGGTGCTGCGCGAAACGGAAAGCGTGTATGATAAACCGCAGGATCAGGTGACGTTTATTGATAACCATGACATTGATCGCTTTAGCCGCAGCGGCCATAGCACGCGTAGCACGGATCTGGGCCTGGCTCTGCTGCTGACTAGCCGTGGCGTGCCGACGATTTATTATGGCACGGAAATCTATATGACGGGCGACGGCGATCCGGATAATCGCAAAATGATGAACACGTTTGATCAGAGCACGGTGGCGTATCAGATTATTCAGCGCCTGTCATCCCTGCGTCAAGAAAATCGCGCGATTGCGTATGGCGATACGACGGAACGCTGGATTAACGAAGATGTGTTTATCTACGAACGCAGCTTTAATGGCGAATATGCGCTGATTGCGGTGAATCGCAATCTGAATCGCAGCTATCAGATTAGCAGCCTGGTGACGGATATGCCGAGCCAGCTGTATGAAGATGAACTGAGCGGCCTGCTGGATGGCCAGAGCATTACGGTGGCGCAGGATGGCAGCGTGCAGCCGTTTCTGCTGGCTCCGGGCGAAGTGAGCGTGTGGCAGTATAGCAATGGCCAGAATGTGGCGCCGGAAATTGGCCAGATTGGTCCGCCGATTGGCAAACCGGGCGACGAAGTGCGTATTGATGGCAGCGGCTTTGGCAATAGCATGGGCAATGTGAGCTTTGCGGGCAGCACGATGAATGTTCTGAGCTGGAACGATGAAACGATTATTGCGGAACTGCCGGTGCATAATGGCGGCAAAAATAGCATTACGGTTACGACGAATAGCGGCGAAAGCAGCAATGGCTATCCGTTTGAACTGCTGACGGGCAGCCAGACGAGCGTGCGCTTTGTGGTGAATCAGGCGGAAACGAGCGTGGGCGAAAATCTGTATCTGGTGGGCAATGTGCCGGAACTGGGCAGCTGGGACCCGGATAAAGCGATTGGCCCGATGTTTAATCAGGTGCTGTATAGCTATCCGACGTGGTATTATGATGTGAGCGTGCCGGCGAATCAGGATATTGAATATAAATATATCATGAAAGATCAGAATGGCAACGTGAGCTGGGAAAGCGGCGGCAATCATATTTATCGCACGCCGGAGAATAGCACGGGCATTGTGGAAGTGAATTATAACCAG

Fig. S1: Codon-optimized sequence encoding for the mature G825-6 CGTase

GGTAACCTGAATAAGGTGAACTTCACCAGCGATATTGTGTATCAGATCGTGGTTGATCGCTTTGTTGATGGCAATACCAGCAATAATCCGAGCGGTGCACTGTTTAGTAGTGGTTGTACCAATCTGCGCAAATATTGTGGCGGTGACTGGCAGGGTATTATTAATAAGATCAACGACGGCTACCTGACCGATATGGGTGTTACCGCAATTTGGATTAGTCAGCCGGTGGAAAATGTTTTTGCAGTTATGAACGACGCGGATGGCAGTACCAGCTATCATGGCTATTGGGCACGCGATTTTAAAAAGACTAACCCGTTTTTCGGTACCCTGAGTGATTTTCAGCGTCTGGTTGATGCAGCCCATGCAAAAGGTATTAAGGTTATTATCGACTTCGCACCGAACCATACCAGTCCGGCCAGCGAAACCAATCCGAGCTATATGGAAAATGGTCGTCTGTATGACAACGGCACCCTGATTGGCGGCTATACCAATGATACCAATAGCTATTTCCACCACAACGGCGGCACCACCTTTAGCAGCCTGGAAGATGGTATCTATCGTAATCTGTTCGACCTGGCCGATTTTAATCATCAGAATCAGTTCATCGACCGCTATCTGAAAGATGCAATTAAGCTGTGGATCGATATGGGCATTGATGGCATTCGTATGGATGCCGTTAAACACATGCCGTTTGGTTGGCAGAAAAGCTTTATGGATGAGATCTATGACTACCGCCCGGTTTTTACCTTTGGTGAATGGTTTCTGAGCGAAAATGAGGTGGATAGTAACAATCACTACTTCGCAAACGAGAGCGGCATGAGTCTGCTGGATTTTCGCTTTGGTCAGAAACTGCGTCAGGTTCTGCGTAATAATAGCGATGATTGGTACGGCTTTAACCAGATGATTCAGGATACCGCCAGTGCATACGATGAAGTTATTGATCAGGTGACCTTCATCGATAACCATGATATGGATCGCTTCATGGCAGATGAAGGCGATCCGCGTAAAGTGGATATTGCACTGGCAGTGCTGCTGACCAGTCGTGGCGTTCCGAATATCTATTATGGTACCGAACAGTACATGACCGGTAATGGTGACCCGAATAATCGTAAAATGATGACCAGTTTCAACAAGAACACCCGTGCATATCAGGTTATTCAGAAACTGAGTAGCCTGCGCCGCAGCAATCCGGCCCTGGCATACGGTGACACCGAACAGCGTTGGATTAATAGTGATGTTTACATCTACGAGCGTCAGTTTGGTAAAGATGTTGTTCTGGTTGCAGTGAATCGTAGTCTGAGCAAAAGCTATAGTATCACCGGCCTGTTTACCGCCCTGCCGAGCGGTACCTATACCGATCAGCTGGGTGGCCTGCTGGATGGTAATACCATTCAGGTGGGTAGTAATGGCAGTGTTAATGCATTCAATCTGGGCCCGGGTGAAGTGGGTGTTTGGACCTATAGCGCCGCAGAAAGTGTGCCGATTATTGGCCATATTGGCCCGATGATGGGCCAGGTGGGTCATAAACTGACCATTGATGGTGAAGGTTTCGGCACCAATGTTGGCACCGTTAAATTTGGCAATACCGTGGCAAGCGTTGTTAGCTGGAGCAATAATCAGATTACCGTTACCGTTCCGAACATTCCGGCAGGCAAATATAATATTACCGTGCAGACCAGTGGCGGTCAGGTTAGCGCAGCCTATGATAATTTCGAAGTTCTGACCAACGACCAGGTGAGCGTTCGCTTTGTTGTTAATAATGCAAACACCAACTGGGGCGAAAATATCTATCTGGTGGGTAATGTTCACGAGCTGGGTAATTGGGATACCAGTAAAGCCATTGGCCCGATGTTTAATCAGGTTATCTATAGTTACCCTACCTGGTATGTGGATGTGAGCGTTCCGGAAGGTAAAACCATTGAATTCAAATTCATCAAGAAGGACGGTAGCGGCAATGTTACCTGGGAAAGCGGCAGTAATCATGTGTATACCACCCCGACCAGCACCACCGGCACCGTTAATGTTAATTGGCAGTAT

Fig. S2: Codon optimized sequence encoding for the mature GeoT-CGTase

A1-Fw 5’-GCGATGGATATCGGAATTAATTCGGATCCGAATGAAAATCTGGATAACGTGAACT

A1-Rev 5‘- GACTGGTATGGTTCGGTGCGAAATCGATGATCACTTTGATGTCATAG

B-Fw 5’- GCCCATGCAAAAGGTATTAAGGTTATTATCGACTTTGTGCCGAATCATACGAGC

B-Rev 5‘-GCATCTTTCAGATAGCGGTCGATGAAGCTATTCTGCTGATTCAGGCT

A2- Fw 5’- CGACCTGGCCGATTTTAATCATCAGAATCAGTTTATCGATCGCTATCTGAAGGAA

A2- Rev 5‘- GGCTACTCAGTTTCTGAATAACCTGATATGCCACCGTGCTCTGATCAAAC

CDE- Fw 5’- ACCAGTTTCAACAAGAACACCCGTGCGTATCAGATTATTCAGCGC

CDE- Rev 5‘- GCTTGTCGACGGAGCTCGAATACTGGTTATAATTCACTTCCACAATG

Fig. S3: Primer sequences selected for domain shuffling. Primer designations refer to the corresponding megaprimer products from the first PCR encoding for G825-6 CGTase domains. G825-6 CGTase encoding regions are marked in grey, vector elements of pET20b(+)::dacD are underlined. Unmarked sequences are derived from the *geoT* fragment and represent target sites for the incorporation of the megaprimer into the vector pET20b(+)::dacD-*geoT* in the second PCR.
